# Registration quality and availability of publications for clinical trials in Germany and the influence of structural factors

**DOI:** 10.1371/journal.pone.0267883

**Published:** 2022-05-09

**Authors:** Christian Thiele, Gerrit Hirschfeld

**Affiliations:** University of Applied Sciences Bielefeld, Faculty of Business, CareTech OWL – Center for Health, Welfare and Technology, Bielefeld, Germany; University of Rennes 1, FRANCE

## Abstract

**Introduction:**

Analyses of clinical trial registries (CTRs) offer insights into methodological problems of published research studies, e.g., non-publication and outcome-switching. Here, we use CTRs as a tool to evaluate clinical studies conducted in Germany and test how their registration quality is associated with time and structural factors: Coordinating Centers for Clinical Trials (KKS) and Universities of Excellence.

**Methods:**

We searched ClinicalTrials.gov, the DRKS, and the ICTRP for clinical trials recruiting participants in Germany. As a measure for the methodological quality, we assessed the proportion of trials that were pre-registered. In addition, the registration quality and availability of publications relating to the trials were manually assessed for a sample (n = 639). Also, the influence of the structural factors was tested using regression models.

**Results:**

We identified 35,912 trials that were conducted in Germany. 59% of trials were pre-registered. Surprisingly, Universities of Excellence had lower pre-registration rates. The influence of KKS was unclear and also difficult to test. Interventional trials were more likely to be pre-registered. Registration quality improved over time and was higher in interventional trials. As of early 2021, 49% of trials that started until the end of 2015 have published scientific articles. 187 of 502 studies on ClinicalTrials.gov for which we found published articles did not reference any in the registry entry.

**Discussion:**

The structural predictors did not show consistent relationships with the various outcome variables. However, the finding that the study type and time were related to better registration quality suggests that regulatory regimes may have an impact. Limitations of this non-pre-registered study were that no modifications to registry entries were tracked and the coarse measure of KKS involvement.

## Introduction

Clinical Trial Registries (CTRs) are an important tool to improve the quality of individual clinical trials and a source of information about the methodological quality of medical research. CTRs, such as ClinicalTrials.gov and the German Clinical Trials Register (DRKS), serve multiple purposes. They serve as public databases for offering trial information to physicians, patients, and potential participants, and they should also foster good scientific practice via the registration of studies.

A large and growing body of literature deals with characteristics of interventional and observational trials, such as prospective registration, registration quality, availability of results, and accompanying scientific articles [1–10]. In a study that analyzed publication rates of articles for clinical trials that German university medical centers ran, it was found that two years after study completion, 39% of trials had published results and six years after study completion, 74% had done so. Additionally, it has been demonstrated that registry data regarding publications is often incomplete, necessitating manual searches for published articles [11]. These results were confirmed in a later cohort [12]. A meta-analysis of 14 methodological research studies that analyzed publication rates of registered trials after a follow-up period of at least 24 months arrived at a pooled estimate of 54.2% of published trials [13].

Another problem that has been uncovered by studies using registry data is discrepancies between registered outcomes and the outcomes that were analyzed and reported [1–3]. While these studies were thus concerned with adherence, a more fundamental requirement, included in the policies of many medical journals, is to pre-register studies [14]. Pre-registration allows for meaningful comparisons between registered and reported outcomes, as in the before-mentioned studies. A meta-analysis has found that the proportion of registered randomized controlled trials has risen from 25% to 52% between 2005 and 2015, whereas only 20% of all trials were pre-registered [4].

For many applications, it is necessary to scrutinize the quality of the CTR entry itself. Viergever and colleagues analyzed a sample of 400 trials registered in 2012 from the International Trials Registry Platform (ICTRP). They found that only about half of the intervention arms were specific in registering the interventions and only 58% of registered outcomes included specific measures and meaningful time-frames [15]. Given that most studies use multiple measures of the same outcome and possibly assess the outcome at different points in time, failure to fully specify the measure or time-frame in the registration increases the researchers’ degrees of freedom.

The present study aims to contribute to the understanding of the mentioned problems and phenomena, explore them further in a German context, and test several structural factors that might influence registration quality or pre-registration rates. The study aims for a broad analysis, as many other studies have focused, for example, on specific medical sub-fields [5–9]. Instead, we started a comprehensive search for both interventional and observational studies with recruiting locations in Germany to gain an overview of registered studies without limiting the search by study topic or study type. This allows for generating broad statistics regarding pre-registration, article availability, and registration quality. An additional aim consists of checking the influence of structural factors specific to Germany, namely the status of a study sponsor as a University of Excellence and the presence of a Coordinating Center for Clinical Trials (KKS) at a trial sponsor’s location.

## Methods

We joined several data sources for subsequent automatic and manual analysis. All data processing and analysis was conducted using R v4.0.3 [16] and the packages tidyverse v1.3.0 [17], lubridate v1.7.9.2 [18], stringr v1.4.0 [19], kableExtra v1.3.1 [20], and modelsummary v0.7.0 [21]. Data and code for reproducing the results are available at https://osf.io/h7exq.

### Data sources

We used three data sources for aggregating trials that had at least one recruiting location in Germany to obtain a data set that is as comprehensive as possible. These were ClinicalTrials.gov as the registry where most older German trials might be registered, the DRKS, which was introduced in 2008 and where most contemporaneous German trials might be registered, and the ICTRP to capture trials from various further registries. The latter is a meta-register aggregating trials from 17 international clinical trial registries, including ClinicalTrials.gov and the DRKS. Data for ClinicalTrials.gov was downloaded from the Aggregate Analysis of ClinicalTrials.gov (AACT).

We joined the three databases and identified duplicates in a step-wise process. First, we joined the primary IDs of ICTRP with the primary IDs of the DRKS and ClinicalTrials.gov, then duplicates without matching IDs that were identified using a Random Forest. The Random Forest used primary IDs, secondary IDs, and other variables, such as the registration date, the titles, the sample sizes, and contact details, as predictors. This model classified 10,537 entries in the ICTRP as internal duplicates and additionally found 26 links between entries from different databases that had not been identified via primary and secondary IDs. That way, we arrived at a dataset of 35,912 trials with a recruiting location in Germany. This approach yielded a merged dataset that was largely free from duplicated studies so that after drawing a sample from it, there were no manual checks necessary for eliminating duplicates. Data from all registries were downloaded during the first weeks of 2021. We used the pipe-delimited files for AACT, the CSV-export function for DRKS, and the full export file in CSV format for the ICTRP. Further details are given in a previous article [22].

For comprehensiveness, we included both interventional and observational trials. Thus, in the context of this study, ‘trial’ may refer to any type of study that was registered in one of the trial databases. Of the 35,912 German studies, we excluded 6 post marketing studies, 32 expanded access studies, two diagnostic test studies, and 1 study of another type using the study type variables because the corresponding trial records often reference other records or material, making it difficult to rate the study record at hand. We drew a random sample of 675 trials, stratified by year, the status of the sponsor as University of Excellence, and sponsor location with a KKS from the overall sample to obtain a substantial number of trials for which published articles would be available. When assessing the quality of registry entries, we did not rely on the extracted data but used the web interfaces of the primary registries, which in the case of the ICTRP refers to the interfaces of the partner registries.

### Automatically extracted study-characteristics

#### Pre-registration

To assess pre-registration, we extracted the registration date at which a trial was entered into the database and the trial’s start date from the databases. These are the variables study_first_submitted_date and start_date in the ‘studies’ table from AACT, created_at, and start_date in DRKS, and Date_registration and Date_enrollment in ICTRP. Since we joined the databases in the previous step, we used the earliest registration date if entries for a single trial were found in multiple databases. As this classification can be done automatically, every trial with non-missing values in the fields as mentioned above can be assessed for pre-registration.

#### Assessment of structural factors

To assess the influence of structural factors on registration quality, namely the influence of the status as University of Excellence and access to a local KKS, we compiled a list of all German Universities of Excellence and the corresponding funding duration and a list of all KKS and their opening years. We used regular expressions to search for the Universities of Excellence in the ‘Primary_sponsor’ variable of the ICTRP, the ‘address.affiliation’ variable of the DRKS, and the ‘name’ variable from the ‘sponsors’ table of the AACT. To compare sponsors that are Universities of Excellence to other university sponsors, we searched the sponsor names for ‘universit’ to capture German and English mentions of university names or university medical centers and labelled the returned studies as having a university sponsor.

There is no information on which trials were supported by a KKS (personal communication with Prof. Dr. Klammt, 2019-02-21). Instead, we flagged trials that a KKS could have potentially supported by considering the KKS’ formal establishment date at the sponsor’s location. The dates when KKS were established at the different locations were taken from the website of the KKS-network [23] and further internet searches. Then we used regular expressions to search for city names in the before mentioned sponsor variables and filtered the returned trials such that the start date of the trial was within the period during which a university received funding via the excellence program or after the opening year of the KKS in the respective city. Again, these classifications can be run automatically for the complete dataset.

### Manual searches

All manual searches and assessments were conducted for the sample of 675 trials that was drawn from the merged database.

#### Manual publication search

**Fig 1:**
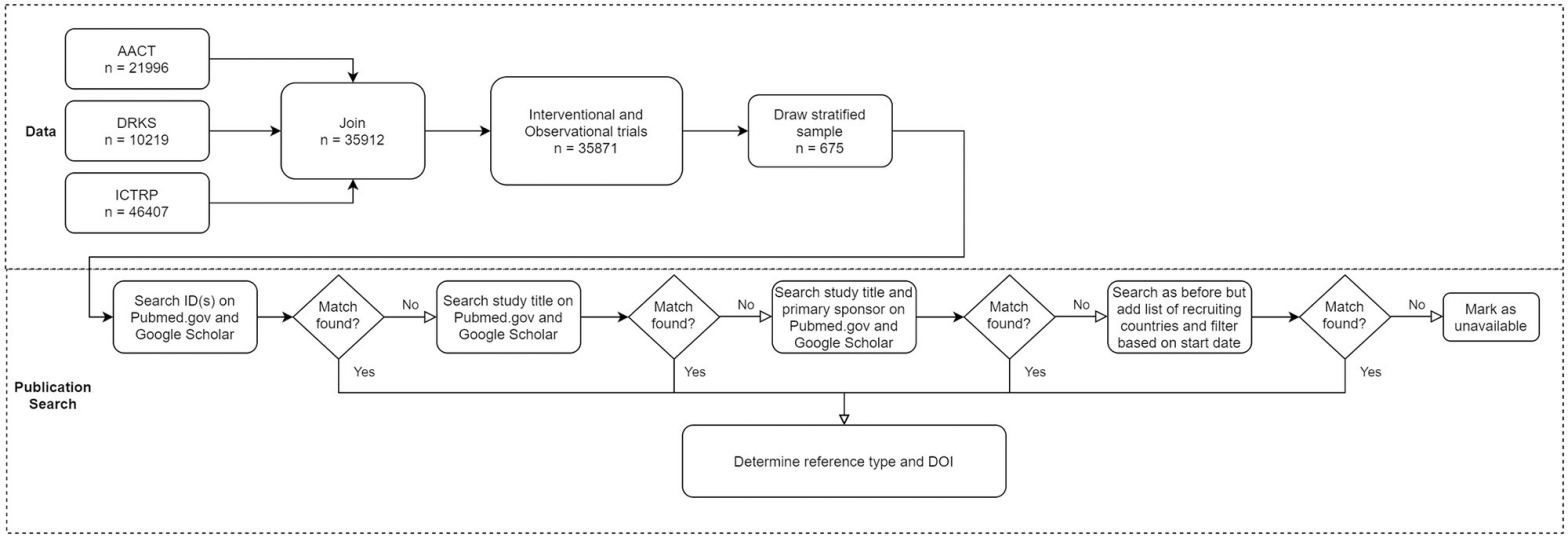
Procedure for data joining and publication search.

We conducted a manual search for publications as follows: We searched the trial ID on Google Scholar and Pubmed, then the study title on Pubmed.gov and Google Scholar. If no matching trials were apparent or the search returned too many similar trials, we narrowed the search by amending the query by the study title and primary sponsor on Google Scholar and Pubmed.gov. As the last step, we searched as before but added recruiting countries and filtered results based on the start date. If a matching full article was found at any of those steps, we aborted the search and added it to our database. If the found reference was something other than a full article, we continued the search (see Fig 1).

#### Manual rating of registration quality

To assess the registration quality, we determined a rating procedure that incorporates quality factors from STROBE [24], CONSORT [25], the WHO Data Set, and previous literature [15]. Eight categories of quality factors were identified: basic study data, study design, outcomes, interventions, analyses, funding, contact details, and result data. These factors consist of multiple pieces of information that should be given in a fully and appropriately registered trial. For example, the details on study design should include a sample size according to all before mentioned sources. We defined rating criteria within all eight major categories, except for the funding, using point rating scales (see Table 1 for examples). We also did not rate result data and contact details, but we searched for scientific publications that contain results, as described above, and tried to contact a subset of responsible parties using contact details from the registries. We used our judgment to determine whether an article presented the actual results of a registered study instead of being a byproduct.

**Table 1:** Examples of database entries and their ratings (AM: Aggregation method).

Category	Points	Example Entry	Comments
Study Design	1	[No internal inconsistency]	
	0	EUCTR2006-001433-17	Parallel and double blind according to title, but registered as non-parallel and open in section E.8
Inclusion Criteria	2	NCT02320045: Body mass index between 22 and 40; Subjects meeting pre-defined estimated glomerular filtration rate criteria and creatinine clearance rate: Normal (≥ 90 mL/min/1.73 m2), Mild (60-89 mL/min/1.73 m2), Moderate (45-59 mL/min/1.73 m2), Moderate (> 30-44 mL/min/1.73 m2)	
	1	NCT01374399: Medical indication: allogeneic stem cell transplantation	Missing description of how the indication is arrived at
	0	NCT01458574: Subjects who met study entry criteria and completed 8-week induction treatment from Study A3921094 or A3921095; Subjects who achieved clinical response in Study A3921094 or A3921095	It would be better to cite the entry and response criteria instead of only referencing other studies
Intervention	2	EUCTR2007-000010-36: Aclidinium Bromide, LAS34273, 200 microgram(s)	Complete (intervention and dosage)
	1	NCT03429543: Start with a low dose of empagliflozin administered once daily and randomly up titrate to the high dose of empagliflozin administered once daily if HbA1c ≥ 7% at week 12	Dosage missing or undefined
	0	NCT01709812: Individualized patient support with compliance supporting tools	Unclear
Outcomes	5	NCT00690898: Percentage of Patients With Relevant Reduction in Pituitary Tumour Volume (as Measured by MRI) From Baseline Volume (Visit 1) to Week 48 (After 12 Injections at Visit 5) [Time Frame: Week 1 and Week 48] […]. A 20% reduction from the volume at Visit 1 was considered to be clinically relevant.	Complete
	4	ISRCTN43578978: Agitation as measured with the Cohen-Mansfield Agitation inventory (CMAI)	No AM (e.g. mean)
	3	NCT01973179: late toxicity [Time Frame: 24 months after therapy] measured from the first day of treatment	No AM (e.g. mean duration), no measure (definition of toxicity)
	2	NCT01012921: The Bone Fill Was Assessed at 6 Months After Regenerative Therapy. [Time Frame: Assessed at 6 months reported]. Change between baseline (regenerative therapy) and the 6 months timepoint is reported.	No type of outcome (e.g. bone gain), no measure (e.g. unit of measurement for bone gain), no AM (e.g. mean)
	1	DRKS00000025: The use of Klacid^®^ will be documented in a high number of patients under conditions simulating practical use. Rare adverse effects shall be traced or previously known good tolerability of the drug shall be confirmed. An additional goal is to collect further data on efficacy and also the rapid onset of action.	no AM, no time frame, no type of outcome and measure (e.g. how efficacy is assessed)
	0	NCT00240214: [missing]	
Title	1	NCT00044915: A Randomized, Double-Blind, Placebo-Controlled Trial to Evaluate the Efficacy, Safety, Tolerability and Pharmacokinetic / Pharmacodynamic Effects of a Targeted Exposure of Intravenous Repinotan in Patients With Acute Ischemic Stroke	Complete
	0	DRKS00004434: Computerised automatic lung CT region-of interest analysis and segmentation	No outcome given

#### Basic study information

We rated the study title and gave one point if all of the intervention, the indication, and the outcome were given. Observational studies did not have to specify an intervention. We did not deduct a point for rather unspecific outcomes such as ‘efficacy’ but deducted a point if the outcome in the title was not one of the primary outcomes.

#### Study design

We rated four criteria concerning the study design. First, we rated the trial design, where we gave one point for an adequate description and zero points if the given information was insufficient or implied internal inconsistencies. An internal inconsistency is, for example, the registration of a study as observational, while the title claims that the study is randomized. Second, we demand a sample size (zero points if not given, one point if given). Third, a description of the masking (zero points if insufficient information was given, one point if there was a description of who was blinded, and two points if the responsible party was also given). We did not rate masking for observational and open-label trials. We regarded the description ‘double blind’ as sufficient for one point. Fourth, the inclusion criteria, where we gave a maximum of two points if diagnoses and accompanying measures were given for all key criteria. Key criteria are all inclusion criteria directly related to the indications and outcomes. For example, a criterion requiring participants to practice acceptable methods of contraception usually has no direct relation to the outcomes and is not rated. We deducted a point if it was not defined how a diagnosis was supposed to be arrived at for all key criteria, e.g., when necessary cutpoints were not given. We gave zero points if the criteria by which patients were screened were unclear.

#### Outcomes and analyses

We rated both primary and secondary outcomes on a five-point scale that had the aim to be as broadly applicable as possible, given the large heterogeneity in our sample of trials. We gave zero points if there was insufficient information and one point each for giving the type of outcome, naming a measure (e.g., BDI-II), giving a time frame, giving a metric, and giving an aggregation method. We regard definitions of change or time of measurement as the metric, for example, ‘change from baseline’ or ‘end value’. Aggregation methods are all statistical procedures for aggregating the individual patient data, for example, a proportion of patients or a mean value across patients. Since we rated both primary and secondary outcomes and some trials have a large number of outcomes, we restricted the number of individual outcomes to rate by rating only the outcome with the most detailed registration.

Additionally, if there were multiple secondary outcomes, we did not rate secondary outcomes that were highly similar to any primary outcome, if possible. If the outcome was survival, we did not deduct a point if the aggregation method was not given, since the standard for this outcome are Kaplan-Meier plots. We also did not deduct a point for the metric criterion if the metric could be inferred from the other pieces of information or if no details on the metric were sensible, for example, if the outcome was simply a diagnosis at a certain point in time.

#### Interventions

We rated the intervention details where zero points were given for insufficient information, one point if the active ingredient or, in the case of non-pharmacological studies, the intervention type was given, and two points if also the dosage or a detailed description of the intervention was given. We did not rate observational studies and control arms for this criterion.

### Survey of trial contacts

We tried to obtain more information regarding released data and cooperation with KKS, so we designed a short questionnaire that we sent out to one contact per trial in our sample. We contacted only individual persons, as opposed to general corporate addresses. Accordingly, one e-mail address per trial was selected manually from the joined trial records if at least one personal e-mail address was present. The e-mail addresses were extracted from the tables ‘central_contacts’ and ‘result_contacts’ in the AACT, from the ‘email’ variable in the DRKS, and from the ‘Scientific_Contact_email’ and ‘Public_Contact_email’ variables in the ICTRP. The questionnaire consisted of only a few questions that asked whether there was cooperation with a KKS and whether results have been published.

### Regression models

We have created linear regression models of varying complexity to predict registration quality by time, involvement of a KKS, involvement of a University of Excellence, study type, registration on ClinicalTrials.gov, and registration on the DRKS. We estimated logistic regression models for pre-registration and article availability using the same independent variables. For pre-registration, we filtered the data for registration dates after 2008 because the DRKS was introduced that year. The registration year and sample size were each standardized by subtracting the mean and dividing by the double standard deviation [26].

## Results

We drew a sample of 675 trials from the overall database of 35871 observational and interventional trials with a recruiting location in Germany. Of the trials in the overall data base 75.69% were interventional and 24.31% were observational. On manual inspection, certain trials were excluded. 8 trials were extension studies, 10 trials were comprised of multiple parts, 13 were patient registry studies, 4 trials were follow-up studies, and 1 trial contained a sub-study. The patient registry studies were excluded as this type of study often only detailed the structure of a specific patient registry. Subsequently, 639 trials were included in the manual analysis.

27.02% of all trials were conducted at one or more sites that have a local KKS and 7.49% were conducted at one or more sites that belong to a University of Excellence at the time of the start of the trial. 5.96% of trials had a local KKS and a University of Excellence. 62% or 29% of all trials are registered on ClinicalTrials.gov and DRKS, respectively (see Table 2). A single trial may be registered on multiple registries, and while ClinicalTrials.gov and the DRKS are among the most important registries for German trials, there are numerous other registries contained in the ICTRP. A full breakdown of the source registries has already been reported previously [22]. The percentages for all of these factors were similar in the sample that was drawn for manual inspection.

**Table 2:** General descriptive information of the overall database and the manually inspected sample.

	Overall		Sample	
	n	%	n	%
n	35871	100	639	1.78
Interventional	27149	75.69	545	85.29
Observational	8722	24.31	94	14.71
Pre-Registered	20794	58.56	364	56.96
Coordinating Center (KKS)	9672	27.02	130	20.34
U of Excellence	2683	7.49	42	6.57
U of Excellence & KKS	2136	5.96	34	5.32
On ClinicalTrials.gov	22306	62.18	502	78.56
On DRKS	10251	28.58	148	23.16
Mean Start Date (SD)	2013-02-03 (2694.75)		2011-04-21 (1881.97)	

### Pre-registration rates

Overall, 58.56% of all trials were pre-registered (see Table 2). Studies without a UoE sponsor or a KKS had the highest pre-registration rate at 62%. Rates for studies with a UoE sponsor or a KKS ranged from 39% to 52% (see Table 3).

**Table 3:** Pre-Registration, registration quality with 95% confidence intervals and article availability in the overall database and in the manually inspected sample depending on structural factors.

	Overall			Sample			
	Pre-Registration			Reg. Quality		Articles	
	n	n	%	n	Mean	n	%
Other	25568	15584	61.73	501	0.78 [0.77-0.8]	215	42.91
KKS	7536	3947	52.38	96	0.73 [0.7-0.75]	42	43.75
U of Exc.	547	211	38.57	8	0.78 [0.68-0.87]	4	50.00
U of Exc. & KKS	2136	1041	48.74	34	0.74 [0.7-0.78]	15	44.12

We also analyzed the association with pre-registration rates of the status as University of Excellence and of KKS for trials registered during the period when Universities of Excellence were funded (2007–2013, see Table 4). We found that trials with a University of Excellence as a sponsor have a lower rate of pre-registration at 43.3%, compared to trials with a sponsor at the location of a KKS with 47.8%. If both a KKS and a University of Excellence were present, 53% of trials were pre-registered. Trials run at sponsor locations without a University of Excellence and a KKS had the highest pre-registration rate at 62.6%. When analyzing only trials with any type of university as their sponsor, the differences are less pronounced with pre-registration rates ranging from 43% to 53%. Here, the highest pre-registration rate is attained by trials that had sponsors at locations with both a University of Excellence and a KKS.

**Table 4:** Summary of logistic regression models of Odds-Ratios for Pre-Registration with p-values in parentheses and 95% confidence intervals.

	Model 1	Model 2	Model 3	Model 4
(Intercept)	1.997 (0.000)	2.692 (0.000)	2.309 (0.000)	2.781 (0.000)
	[1.945, 2.050]	[2.497, 2.902]	[2.239, 2.382]	[2.577, 3.002]
U of Excellence	0.459 (0.000)	0.471 (0.000)		
	[0.421, 0.500]	[0.429, 0.517]		
Registration Year		0.867 (0.000)		0.978 (0.422)
		[0.819, 0.917]		[0.925, 1.033]
Sample Size		1.031 (0.556)		1.034 (0.513)
		[0.932, 1.140]		[0.936, 1.142]
Observational		0.446 (0.000)		0.449 (0.000)
		[0.422, 0.473]		[0.424, 0.475]
On ClinicalTrials.gov		1.170 (0.000)		1.148 (0.000)
		[1.084, 1.264]		[1.063, 1.240]
On DRKS		0.673 (0.000)		0.700 (0.000)
		[0.622, 0.728]		[0.646, 0.758]
KKS			0.521 (0.000)	0.723 (0.000)
			[0.494, 0.549]	[0.682, 0.766]
Num.Obs.	27523	27106	27523	27106
AIC	35307.9	33186.8	35032.6	33323.0
BIC	35324.3	33244.2	35049.1	33380.4
Log.Lik.	-17651.926	-16586.394	-17514.311	-16654.481
RMSE	1.13	1.11	1.13	1.11

Inspecting the results of the logistic regression models for pre-registration (see Table 5), we found that the registration year, which was included to capture possible time trends, showed a decrease in studies that are pre-registered in the model including UoE status (OR = .87; 95% CI = .82-.92; p <.001) but was insignificant in the model including KKS. Observational trials were less likely to be pre-registered (OR = .45; p <.001). There were differences depending on the registry, with trials registered on ClinicalTrials.gov being more likely to be pre-registered (OR = 1.17; 95% CI = 1.08-1.26; p <.001) and trials on DRKS being less likely to be pre-registered (OR = .67; 95% CI = .62-.73; p <.001). The structural predictors implied that having a location with a KKS was associated with lower pre-registration rates (OR = .72; 95% CI = .68-.77; p = <.001). Similarly, UoE-status was also associated with lower pre-registration rates (OR = .47; 95% CI = .43-.52; p = <.001).

**Table 5:** Mean quality ratings in Clinicaltrials.gov, DRKS, and other registries, grouped by study type (Int: Interventional, Obs: Observational).

Criterion	ClinicalTrials.gov		DRKS		Other	
	Int	Obs	Int	Obs	Int	Obs
Design	0.98	0.96	0.95	1	0.96	1
Inclusion Criteria	1.77	1.45	1.69	1.65	1.87	1.71
Interventions	1.69		1.53		1.85	
Primary Outcome	3.97	3.17	3.62	2.78	3.59	2.43
Secondary Outcome	3.88	3.24	3.02	1.92	2.81	2.14
Sample Size	1	1	1	1	0.98	1
Blinding	0.99		1.03		0.77	
Title	0.74	0.57	0.75	0.58	0.76	0.57

### Registration quality

We fit four linear regression models to predict the registration quality score using the same predictors as before (see Table 6). We found an overall positive trend for the registration year, indicating that the registrations are improving overall (beta = .08; 95% CI = .06–.10, p <.001). Also, trials registered on ClinicalTrials.gov had a higher registration quality than those registered on other databases (beta = .08; 95% CI = .05–.10, p <.001). Observational trials had a lower registration quality than other trials (beta = -.10; 95% CI = -.13–-.08, p <.001). Regarding the structural predictors, we found that the involvement of a KKS was associated with lower registration quality (beta = -.03; 95% CI = -.05–.00, p <.05). There was no significant association between UoE status and registration quality.

**Table 6:** Summary of linear regression models predicting mean percentage of attainable registration quality score over all criteria with P-Values in parentheses and 95% confidence intervals.

	Model 1	Model 2	Model 3	Model 4
(Intercept)	0.72 (0.00)	0.67 (0.00)	0.72 (0.00)	0.68 (0.00)
	[0.71, 0.73]	[0.65, 0.70]	[0.71, 0.73]	[0.66, 0.70]
U of Excellence	-0.02 (0.38)	0.00 (0.94)		
	[-0.06, 0.02]	[-0.04, 0.03]		
Registration Year		0.08 (0.00)		0.08 (0.00)
		[0.06, 0.10]		[0.06, 0.10]
Sample Size		0.00 (0.83)		0.00 (0.97)
		[-0.02, 0.02]		[-0.02, 0.02]
Observational		-0.10 (0.00)		-0.10 (0.00)
		[-0.13, -0.08]		[-0.13, -0.07]
On ClinicalTrials.gov		0.08 (0.00)		0.07 (0.00)
		[0.05, 0.10]		[0.05, 0.10]
On DRKS		-0.01 (0.33)		-0.01 (0.49)
		[-0.03, 0.01]		[-0.03, 0.02]
KKS			-0.04 (0.00)	-0.03 (0.02)
			[-0.07, -0.02]	[-0.05, 0.00]
Num.Obs.	639	638	639	638
R2	0.001	0.217	0.021	0.223
R2 Adj.	0.000	0.209	0.019	0.216
AIC	-853.9	-996.8	-866.5	-1001.9
BIC	-840.5	-961.1	-853.1	-966.2
Log.Lik.	429.951	506.408	436.261	508.958
F	0.768	29.093	13.489	30.171
RMSE	0.12	0.11	0.12	0.11

**Fig 2:**
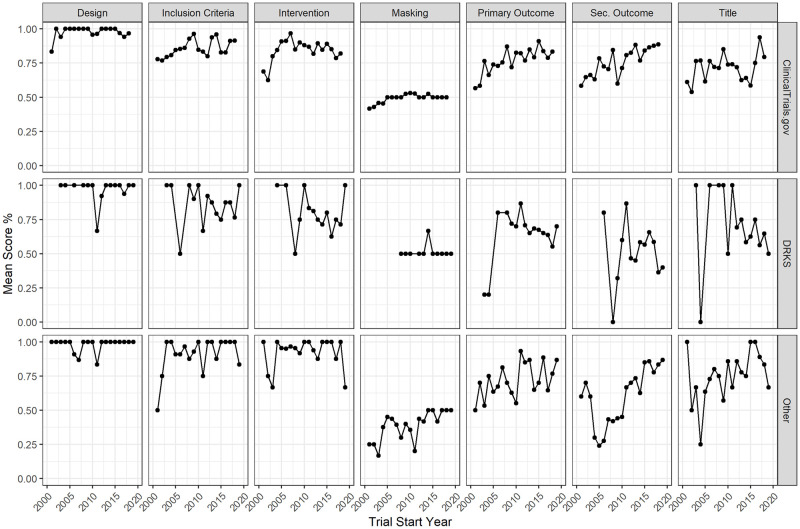
Trends in registration quality over time separately for the different registries.

We also inspected the registration quality of the specific criteria across the different registries. The temporal trends for the individual criteria are depicted as mean quality scores per year and criterion in Fig 2, separately for ClinicalTrials.gov, the DRKS, and further registries imported via the ICTRP. The assessment of the registration quality of sample sizes is left out here because we found only three trials not to report a sample size. While we have seen a generally positive trend over time, this is mostly true for ClinicalTrials.gov and for the other registries imported via the ICTRP, for which we find positive time trends for all criteria when regressing the trial start year on the mean registration quality per criterion. The start year coefficients are significant (*p* <.05) for 3 out of 7 criteria in the case of ClinicalTrials.gov and 5 out of 7 criteria of other registries from ICTRP. The trends are not as evident for the DRKS with a positive coefficient of the trial start year only for the registration quality of the trial design and no coefficient being significant (results not shown). Apart from temporal trends, the mean registration quality was higher for ClinicalTrials.gov in most categories compared to the DRKS. Registration qualities of the inclusion criteria, interventions, and the primary outcomes were similar between ClinicalTrials.gov and other registries from the ICTRP (see Table 7).

**Table 7:** Number and percent of Pre-Registered studies depending on status as university of excellence and on presence of a coordinating center for clinical studies. Trials that were registered between 2007 and 2013.

Group	Overall			Only Universities		
	Pre-Registered			Pre-Registered		
	n	n	%	n	n	%
KKS	1549	740	47.77	1248	595	47.68
Other	10360	6483	62.58	1271	588	46.26
U of Exc. & KKS	1236	655	52.99	1236	655	52.99
U of Excellence	404	175	43.32	404	175	43.32

### Article availability

We manually searched for scientific articles that report trial results. We found that 43% of all trials had an associated article in our sample. We compared the manually found articles to the references given in the 502 studies on ClinicalTrials.gov. 52 studies referenced at least one published article that reported the results of the respective study. We found the same article on ClinicalTrials.gov as the one that was found manually in 30 trials, articles for 10 trials for which we had found none manually, 12 trials with other articles in addition to the manually found one, and 8 trials referenced related articles that did not report results of the trial registration at hand. 263 trials for which we found no published articles did not reference any articles on ClinicalTrials.gov as well, whereas 187 articles for which we had found published articles manually did not reference any articles on ClinicalTrials.gov (Table 8).

**Table 8:** Number of studies for which articles could be found manually and a comparison of those articles to the references found on ClinicalTrials.gov, also as number of studies.

Any Article Found Manually	References on ClinicalTrials.gov			
	Same	Additional	Related	None
Yes	30	12	5	187
No	0	10	3	263

**Fig 3:**
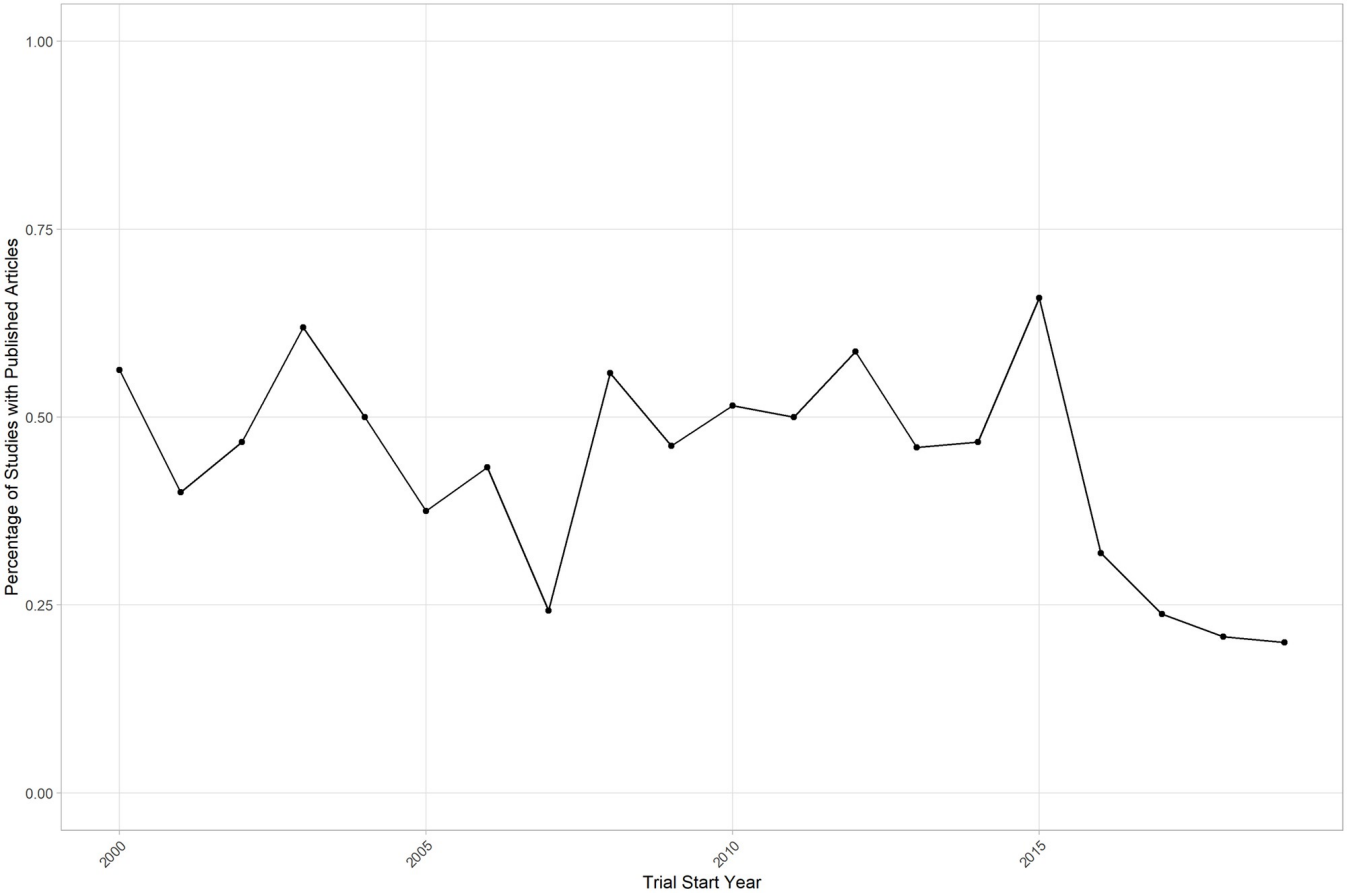
Percentage of trials with published scientific articles in the manually inspected sample.

When running the before mentioned regression models again for article availability as the dependent variable, we find no significant associations between the predictors and the criterion variables. Numerically, the coefficient for the registration year is negative, which was expected, given the delay between trial registration and article publication, but is also insignificant (see Table 9). Of all trials that started in 2018 or later, 21% have published articles, whereas this estimate is 49% for all trials that started until the end of 2015. The percentage of trials with published articles is relatively stable over time, except for trials started in 2015 or later, where the percentage gradually decreases due to the time needed for completion of the trial and publication of results (see Fig 3).

**Table 9:** Summary of logistic regression models of Odds-Ratios for article availability with p-values in parentheses and 95% confidence intervals.

	Model 1	Model 2	Model 3	Model 4
(Intercept)	0.761 (0.001)	0.517 (0.003)	0.761 (0.002)	0.510 (0.003)
	[0.647, 0.895]	[0.334, 0.803]	[0.639, 0.907]	[0.325, 0.799]
U of Excellence	1.195 (0.578)	1.216 (0.546)		
	[0.638, 2.236]	[0.645, 2.291]		
Registration Year		0.764 (0.114)		0.757 (0.103)
		[0.548, 1.066]		[0.542, 1.058]
Sample Size		0.971 (0.861)		0.976 (0.886)
		[0.697, 1.352]		[0.700, 1.361]
Observational		1.357 (0.206)		1.340 (0.229)
		[0.845, 2.180]		[0.832, 2.159]
On ClinicalTrials.gov		1.461 (0.090)		1.475 (0.085)
		[0.942, 2.267]		[0.948, 2.294]
On DRKS		1.159 (0.497)		1.151 (0.520)
		[0.757, 1.776]		[0.750, 1.768]
KKS			1.058 (0.775)	1.126 (0.573)
			[0.718, 1.559]	[0.745, 1.702]
Num.Obs.	639	638	639	638
AIC	878.7	880.4	878.9	880.5
BIC	887.6	911.7	887.9	911.7
Log.Lik.	-437.361	-433.224	-437.474	-433.247
RMSE	1.17	1.17	1.17	1.17

### Results of the survey of trial contacts

To not only rely on automatic classification of study locations and our search of publications, we sent out 285 invitations to a questionnaire, which asked responsible researchers about cooperation with a KKS and publication of results. 64 invitational e-mails could not be delivered, because the e-mail addresses were not active anymore. Of 221 invited researchers, 47 finished the questionnaire. 38 of these were responsible for trials registered on the DRKS.

35 researchers replied that the results of the trial in question were presented at a conference or published in a scientific journal. Of these, 27 also supplied a Pubmed-ID or DOI. Compared to the manually searched references and the ones contained in the AACT, 11 of those 27 articles had been found, 12 were not found, and 4 were additional articles from trials for which other articles had been found.

Seven researchers said that they cooperated with a KKS for the respective trial. Of these, two did so after enrollment had started. Of the seven trials for which cooperation with a KKS was indicated, three had a sponsor location with a KKS. 18 trials for which we have received responses had at least one location with a KKS, but did not indicate cooperation with a KKS. Four trials did not have a KKS at a sponsor’s location, but cooperation with a KKS was still indicated.

## Discussion

After analyzing data on registration quality, article availability, survey responses, and pre-registration rates, some patterns have emerged, most of which are in line with estimates from previous research. We arrived at a proportion of close to 50% of trials that have published results six years after the trial started, both in interventional and observational trials. This proportion is about 20% lower than estimates from other studies [11]. However, we have not narrowed down our trial selection, including trials that have not been marked as finished and trials from all sponsor types. It seems justified not to exclude these trials because we have found, for example, 11 articles for studies with completion dates until the end of 2020 and the status ‘unknown’. Thus, we believe we have arrived at an estimate that describes publication rates very broadly. This estimate of 50% is also close to the proportion of positive responses from our survey. The survey estimate of publication rates is a few percentage points higher, which would be expected, though, due to adverse selection when only considering completed questionnaires. Additionally, 44% of the survey responses referenced articles that had not been found manually. Given that 81% of the survey responses came from studies registered on the DRKS and the regression results hinting at lower publication rates for the DRKS, this may merely imply more difficult discoverability of publications for DRKS trials, but more data would be needed to confirm that.

Regarding registration quality, we could successfully apply our rating scheme to a wide range of trials. Such a broadly applicable scheme is not designed to capture more minor flaws in trial registrations, which should be left to studies of specific medical fields. However, some patterns in registration quality have emerged, suggesting that the rating scheme did indeed capture at least some of the systematic differences and quality characteristics. The time trend in registration quality was generally positive, which is in line with previous research [15]. Nevertheless, there were notable differences between ClinicalTrials.gov and the DRKS. On average, ClinicalTrials.gov attained higher ratings and showed a positive time trend in registration quality more consistently. First, this may be due to the way the rating scheme was designed, as we can not rule out that it systematically gave more weight to pieces of information that are of higher registration quality in ClinicalTrials.gov while neglecting others. Second, these rating differences may also be caused by the way the registries enable data entry via web forms and the structure of those forms, as this may ‘nudge’ researchers to be more or less specific about certain trial details. For example, records from EUCTR always gave dosages of their interventions.

No clear patterns emerged regarding the influence of German structural factors, namely whether a trial sponsor was close to a Coordinating Center for Clinical Trials and whether one of the sponsor locations had the status as University of Excellence at trial registration. It seemed that trials with these characteristics had lower registration quality and were less often pre-registered than other trials, in some cases significantly, depending on the models’ control variables. For example, having a sponsor close to a KKS lead to 3-4% lower mean registration quality. However, we are cautious in drawing conclusions here because the limited data from survey responses suggested that automatic determination of the KKS-factor may not be feasible, at least not if the effect of cooperating with a KKS should be investigated. Much larger survey data would be needed to arrive at a sufficient sample size for re-analyzing that factor.

The DRKS was introduced to collect the previously very incomplete data of German clinical studies in a central database and to facilitate analyses of clinical research in Germany. Another goal was to simplify the work of clinical researchers by involving ethics committees, which already had to approve all clinical trials in Germany [27]. The coordination centers initially funded by the BMBF pursue similar goals in that clinical research in Germany should be professionalized. Unfortunately, notwithstanding any improvements in support for researchers, our results indicate that this did not also translate into markedly improved registration of studies. To this end, coordinating centers, the DRKS, and researchers may strive to intensify their cooperation.

In summary, the results of the present study need to be interpreted in light of several limitations. First, we had to rely on very coarse measures of KKS-involvement. While it could be argued that this is the level at which governmental interventions into the research system operate, it leaves open the possibility that individual trials benefit by involving KKS. Furthermore, KKS may of course also support studies that are sponsored at locations different from their own one. Secondly, we only inspected the latest version of each trial record instead of the first or intermediate versions. The reason for this was that we assumed that authors usually update the trial record so that the latest version should be the most accurate one. Lastly, this study was not pre-registered, but it was outlined in the accompanying grant. Thus, and because of the limited data for studies with involvement of UoE or KKS, the results should be interpreted as descriptive, rather than as causal effects.

Overall, the present study confirms earlier findings regarding the quality of study-registrations [15]. Flaws in trial registrations are a hurdle for studies into the adherence of published research findings because only if the outcomes are described in sufficient detail CTRs can provide an added level of methodological rigor. While our analysis of broad structural factors did not show a consistent impact of these interventions, the comparison of different study types shows significant differences. Therefore, we believe that future interventions should start with a more fine-grained analysis of these differences.
